# Indirect Mortality from Influenza

**Published:** 1890-06-14

**Authors:** 


### INDIRECT MORTALITY FROM INFLUENZA.

That the deaths certified and registered as caused by influenza by no means indicate the actual loss of life attending an epidemic like that of last winter was clearly shown by Dr. Jacques Bertillon, head of the Statistical Department of the Municipality of Paris, in an address delivered by him before the Epidemiological Society. In that city the deaths returned under influenza during the months of December and January were only 213, whereas in the same period the total mortality or deaths from all causes was 5,500 above the average. For this no cause other than the epidemic of influenza could be assigned, since there was no greater prevalence of infectious disease, and the winter was, if anything, less severe than usual.

The enormous increase in deaths from pneumonia, pleurisy, and bronchitis, Dr. Bertillon believes to represent cases of influenza in which medical advice was not sought until those conditions had supervened as sequelæ of the primary disease, which thus did not appear in the certificates, while the increase in the mortality from phthisis, organic lesions of the heart, diabetes, alcoholism, and chronic diseases of the brain, kidneys, &c., he explains by the super-addition of influenza causing or accelerating a fatal termination which might otherwise have been postponed for months, or even years. A large number of these deaths occurred suddenly, probably from implication of the ganglia of the heart.

Dr. Wiltchur, of St. Petersburg, makes the same observation with regard to phthisis, the type of which, when complicated with influenza, was sharply distinguished from that commonly met with. The patients, he says, were for the most part still well nourished. Cyanosis of the face and extremities was frequent, with asthma, extreme prostration, and temperature of 105° F. or more. The progress of the phthisical disease was rapid, and pulmonary hæmorrhages occurred frequently. Many cases died most unexpectedly. He also noticed the frequent development during an attack of influenza, of rapid phthisis (galloping consumption) in persons who had not previously shown any indications of weakness of the lungs.

All attempts at the discovery of a specific or characteristic microbe have as yet been fruitless, Levy, Finkler, Klebs, Weichselbaum, and other trustworthy observers and experienced bacteriologists having succeeded in identifying only those such as Fränkel's *Diplococcus pneumoniæ*, the *Staphylococcus aureus*, and *Streptococcus pyogenes*, which occur in all catarrhal conditions of the bronchial and pulmonary mucous membranes.

